# Perivascular Epithelioid Cell Tumor of Gastrointestinal Tract: Case Report and Review of the Literature

**DOI:** 10.1097/MD.0000000000000393

**Published:** 2015-01-26

**Authors:** Biyan Lu, Chenliang Wang, Junxiao Zhang, Roland P. Kuiper, Minmin Song, Xiaoli Zhang, Shunxin Song, Ad Geurts van Kessel, Aikichi Iwamoto, Jianping Wang, Huanliang Liu

**Affiliations:** From the Guangdong Institute of Gastroenterology and the Sixth Affiliated Hospital, (BL, CW, JZ, MS, XZ, SS, JW, HL); Guangdong Key Laboratory of Colorectal and Pelvic Floor Diseases, (BL, CW, JZ, MS, XZ, JW, HL); Institute of Human Virology, (BL, CW, JZ, MS, XZ, HL) Key Laboratory of Tropical Disease Control (Ministry of Education); Sun Yat-sen University, Guangzhou (BL, CW, JZ, MS, XZ, HL); Dongguan Health School, Dongguan, China (BL); Department of Human Genetics, Radboud University Medical Center, Nijmegen, The Netherlands (JZ, RPK, AGK); and Advanced Clinical Research Center, Institute of Medical Science, University of Tokyo, Tokyo, Japan (AI).

## Abstract

Perivascular epithelioid cell tumors of gastrointestinal tract (GI PEComas) are exceedingly rare, with only a limited number of published reports worldwide. Given the scarcity of GI PEComas and their relatively short follow-up periods, our current knowledge of their biologic behavior, molecular genetic alterations, diagnostic criteria, and prognostic factors continues to be very limited.

We present 2 cases of GI PEComas, one of which showed an aggressive histologic behavior that underwent multiple combined chemotherapies. We also review the available English-language medical literature on GI PEComas-not otherwise specified (PEComas-NOS) and discuss their clinicopathological and molecular genetic features.

Pathologic analyses including histomorphologic, immunohistochemical, and ultrastructural studies were performed to evaluate the clinicopathological features of GI PEComas, their diagnosis, and differential diagnosis. Immunohistochemistry, semiquantitative reverse transcriptase polymerase chain reaction, and DNA sequencing assays were carried out to detect the potential molecular genetic alterations in our cases

Microscopically, the tumors showed distinctive histologic features of PEComas-NOS, including fascicular or nested architecture, epithelioid or spindled cell type, and clear to eosinophilic cytoplasm. The tumor cells were immunohistochemically positive for melanocytic markers. Molecular pathological assays confirmed a *PSF-TFE3* gene fusion in one of our cases. Furthermore, in this case microphthalmia-associated transcription factor and its downstream genes were found to exhibit elevated transcript levels.

Knowledge about the molecular genetic alterations in GI PEComas is still limited and warrants further study.

## INTRODUCTION

Perivascular epithelioid cell tumors (PEComas) are a family of rare mesenchymal neoplasms histologically and immunohistochemically characterized by perivascular epithelioid cell (PEC) differentiation.^1^ The PECs have variable morphologic features, with an epithelioid to spindled cell type resembling smooth muscle, clear to granular lightly eosinophilic cytoplasm, and round to oval nuclei with small nucleoli. The PECs also exhibit a distinct immunophenotype with a coexpression of melanocytic and myogenic markers, such as HMB45, Melan-A, MiTF, smooth muscle actin (SMA), and calponin.^2^ The PEComa family includes angiomyolipoma (AML), clear cell “sugar” tumor of the lung (CCST), lymphangioleiomyomatosis (LAM), clear cell myomelanocytic tumor of the falciform ligament/ligamentum teres (CCMMT), and unusual clear cell tumors in other locations. PEComas have been reported in various anatomic sites, with a marked female predominance. Due to their relative rarity, the diagnostic criteria, optimal treatment strategies, and prognostic factors for PEComas have not yet been confirmed at this time. We report 2 cases of PEComas arising in the gastrointestinal tract, including the clinicopathological features and potential molecular genetic alterations of this rare tumor.

## MATERIALS AND METHODS

### Case Presentation

#### Case 1

**FIGURE 1:**
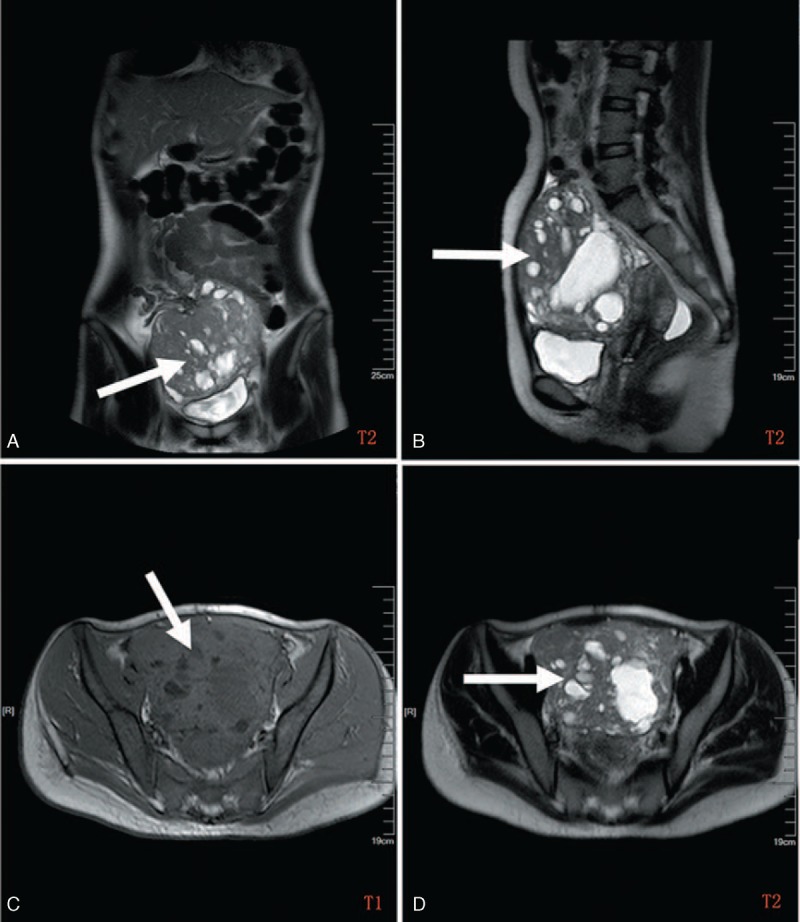
Abdominal computed tomography images (case 1): coronal (A), sagittal (B), and axial (C and D) reconstructions. Computed tomography of the abdomen showed an ill-defined multilocular low-density mass measuring 13 × 8 × 7 cm in the pelvis and lower abdomen.

A 29-year-old Chinese woman was admitted to our hospital because of gradual onset of abdominal pain, nausea, vomiting and weight loss for 6 months.^3^ The patient did not have a medical history of gastrointestinal tumors, inflammatory bowel disease, or tuberous sclerosis complex. Her family history was unremarkable. Physical examination revealed a large mass at the right lower abdomen. All blood and biochemical tests were within the normal ranges, apart from a hemoglobin reading of 85 mg/dL and a C-reactive protein (CRP) reading of 15 mg/dL. An intravenous contrast-enhanced computed tomography (CT) scan showed an ill-defined multilocular soft tissue tumor measuring 13 cm × 8 cm × 7 cm in the pelvis and lower abdomen (Figure 1). During the surgical operation, a large tumor was found in the terminal ileum about 12 cm from ileocecal valve adhering tightly to the mesentery of the ileum and the right pelvic wall. Surgical resection of the tumor and the affected segment of the intestine was carried out. After surgery, the patient received 5 courses of multiple combined chemotherapies including ifosfamide 2000 mg/m^2^ day 1 to 4, epidoxorubicin 30 mg/m^2^ day 1 to 3, dacarbazine 350 mg/m^2^ day 1 to 4, and mesna 4800 mg/m^2^ day 1 to 4 once every 3 weeks (Q3W). In addition, granulocyte colony-stimulating factor (G-CSF; Filgrastim) was given at a dose of 5 pg/kg/day subcutaneously from day 5 to day 12 of each cycle. Follow-up CT scans were performed every 6 months after chemotherapy. The patient was alive and well with no signs of recurrence or metastasis for 28 months of follow-up.

#### Case 2

**FIGURE 2:**
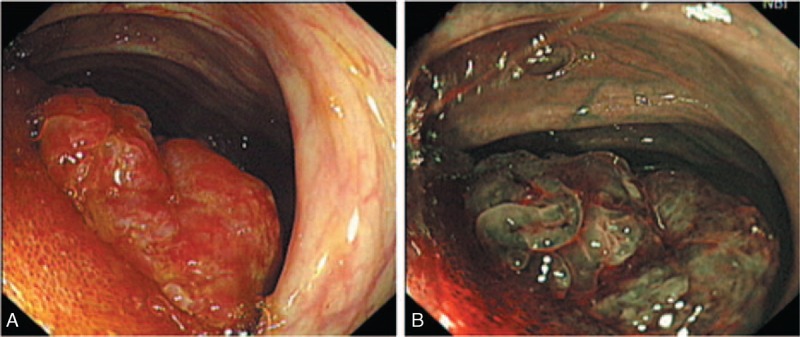
Endoscopy appearance of GI PEComa (case 2). White light (A) and narrow band imaging (B) endoscopy showed a 2-cm diameter, polypoid tumor protruding into the lumen of the terminal ileum. GI PEComa = perivascular epithelioid cell tumors of gastrointestinal tract.

A 41-year-old Chinese woman with a history of hysterectomy for benign leiomyoma presented with progressive epigastric pain and dark stools. The patient denied any family history of gastrointestinal (GI) cancer or inflammatory bowel disease. Laboratory investigations showed a white blood cell (WBC) count of 11.6 × 10^9^ cells/L with 87.3% neutrophils and a hemoglobin reading of 100 mg/dL. Serum levels of CA19-9, CEA, AFP, and CA125 were within normal limits. The abdominal CT scan and ultrasonography revealed ileocecal intussusception with a tumor in the ileum. An enteroscopy displayed a 2-cm diameter, polypoid, submucosal tumor in the terminal ileum (Figure 2). At laparotomy, neither celiac lymphadenectasis nor distant metastatic foci were detected. Approximately, 45 cm of the terminal ileum (20 cm) and proximal colon (25 cm) were resected. Without additional therapy, the patient remained asymptomatic and free of disease at 39 months postoperatively.

### Histologic and Immunohistochemical Analyses

The tumor tissues were fixed in 10% phosphate buffered saline formalin solution and embedded in paraffin. Tissue sections were stained with hematoxylin and eosin for microscopic examination. Immunohistochemical stainings were performed with the primary antibodies listed in Table 1 by using the DAKO Envision detection kit (Dako Cytomation, Carpinteria, CA), according to standard procedures. The Ki-67 labeling index was defined as the percentage of MIB-1-positive cells among at least 500 tumor cells in 5 representative fields. This study was approved by the Committee of the Institute of Research and Medical Ethics at Sun Yat-Sen University.

**TABLE 1:**
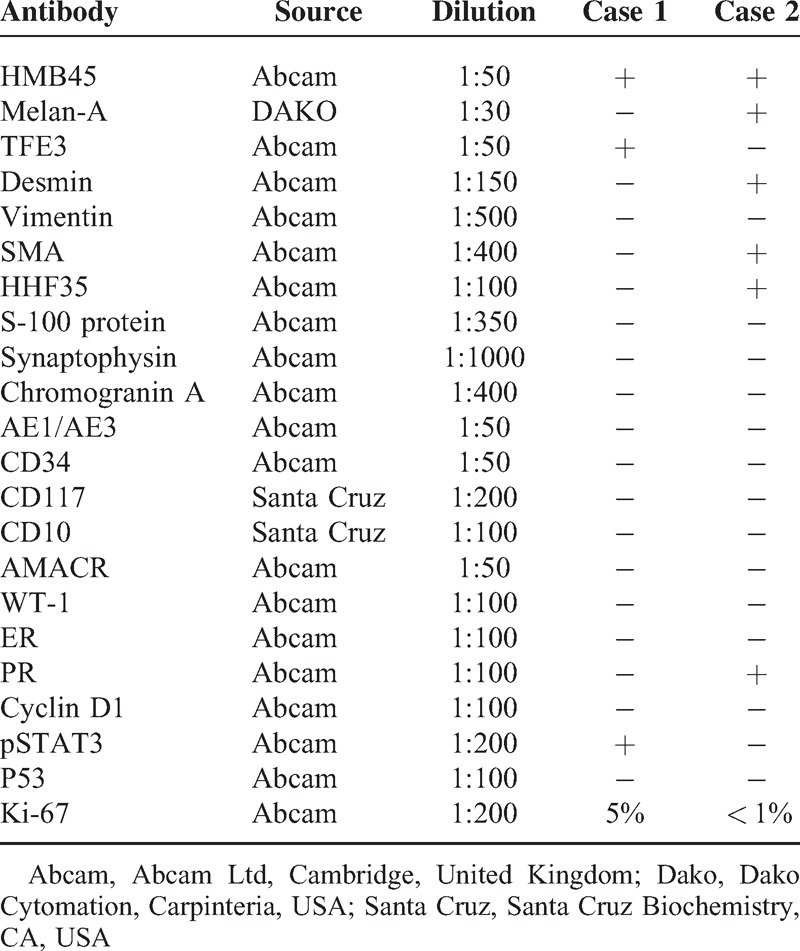
Immunohistochemical Results of the 2 Cases

### Electron Microscopy

The tumor samples were collected and fixed in 4% phosphate-buffered solution of glutaraldehyde and osmium tetroxide. Ultrathin sections were cut for ultrastructural evaluation using a Tecnai G^2^ Spirit TWIN electron microscope (FEI Co, Eindhoven, Netherlands).

### Reverse Transcription-Polymerase Chain Reaction and Sequencing

A reverse transcription-polymerase chain reaction (RT-PCR) assay was performed to detect related gene fusions. Total RNA was isolated from the tumor tissues using TRIzol reagent (Invitrogen, Carlsbad, CA) according to the manufacturer's instructions. The first strand of cDNA was obtained using RevertAid First Strand cDNA Synthesis Kit (MBI Fermentas, Vilnius, Lithuania). PCR was performed with rTaq polymerase (Takara Shuzo, Ohtsu, Japan). The PCR primers and cycling conditions were performed as described in Table 2. The resulting PCR products were analyzed in 1.5% agarose electrophoretic gels using DL1, 000 DNA Marker (Takara Shuzo) as a size reference. Gel images were obtained with Gene Genius (Syngene, Frederick, MD). The specific PCR product was sent to Shanghai Invitrogen Biotechnology Co., Ltd (Guangzhou Office) for purification and sequencing. In the analysis of MiTF and its downstream genes expression, malignant melanoma tissue served as a positive control, and normal intestinal mucosa distant from cancer served as a negative control.

**Table 2:**
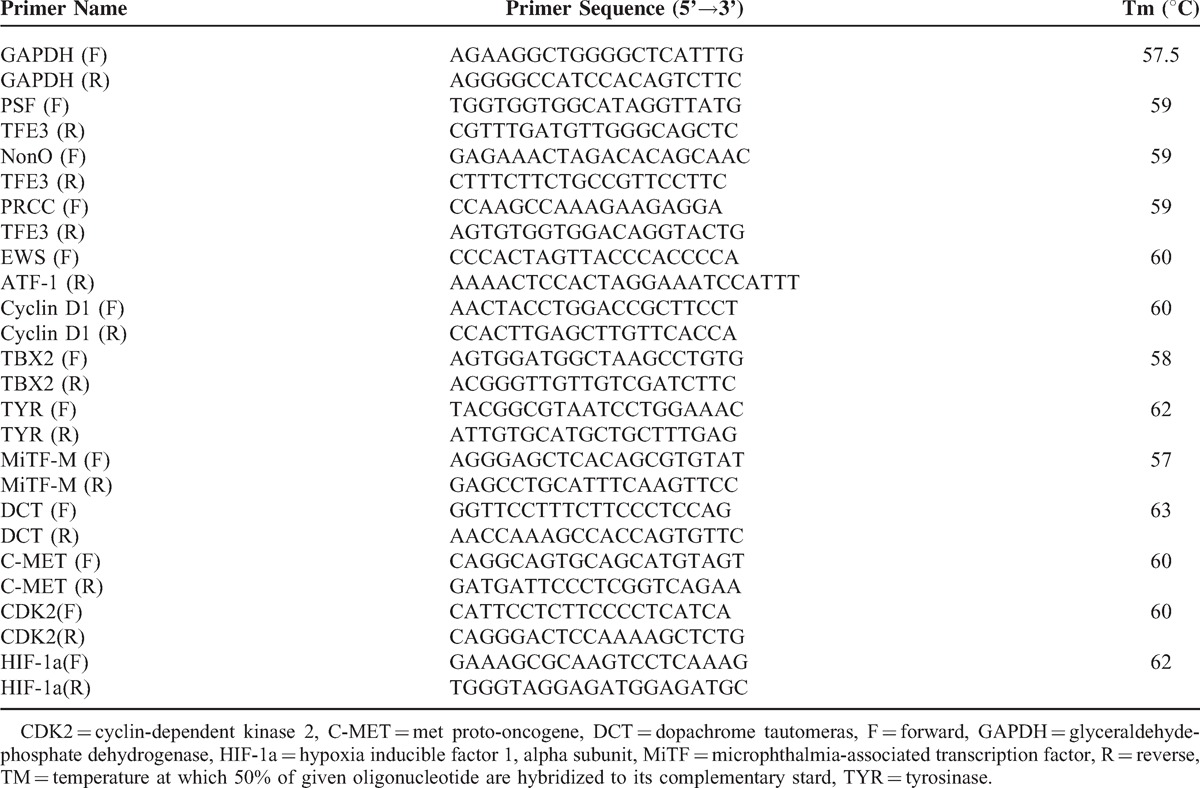
Details of Primers Used in the Study

## RESULTS

### Pathologic Findings

#### Case 1

**FIGURE 3:**
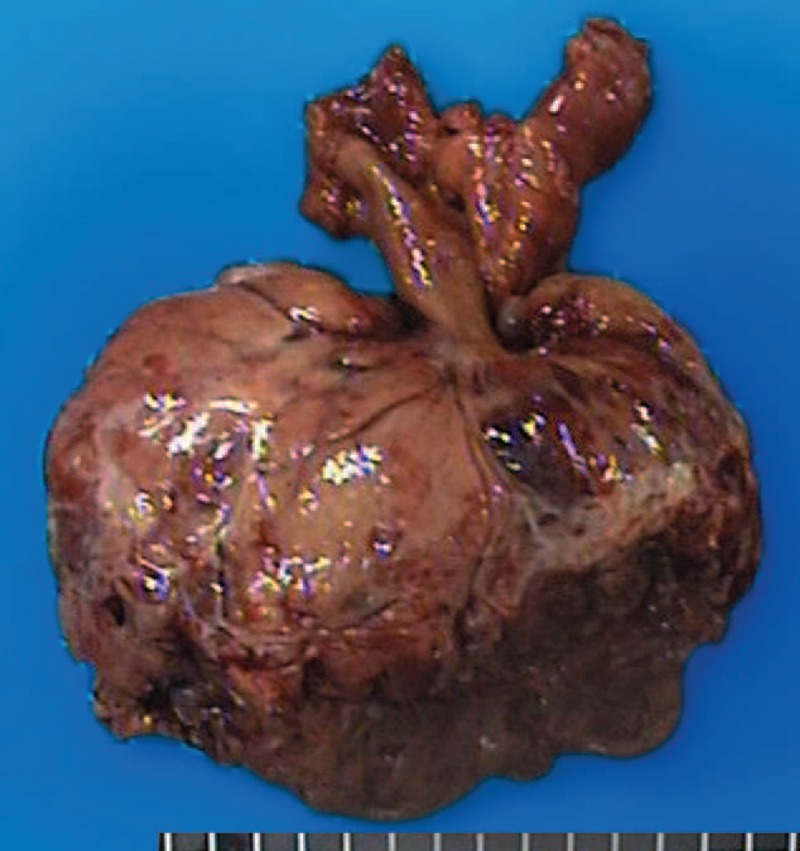
Resected specimens of perivascular epithelioid cell tumor arising in the terminal ileum (case 1).

**FIGURE 4:**
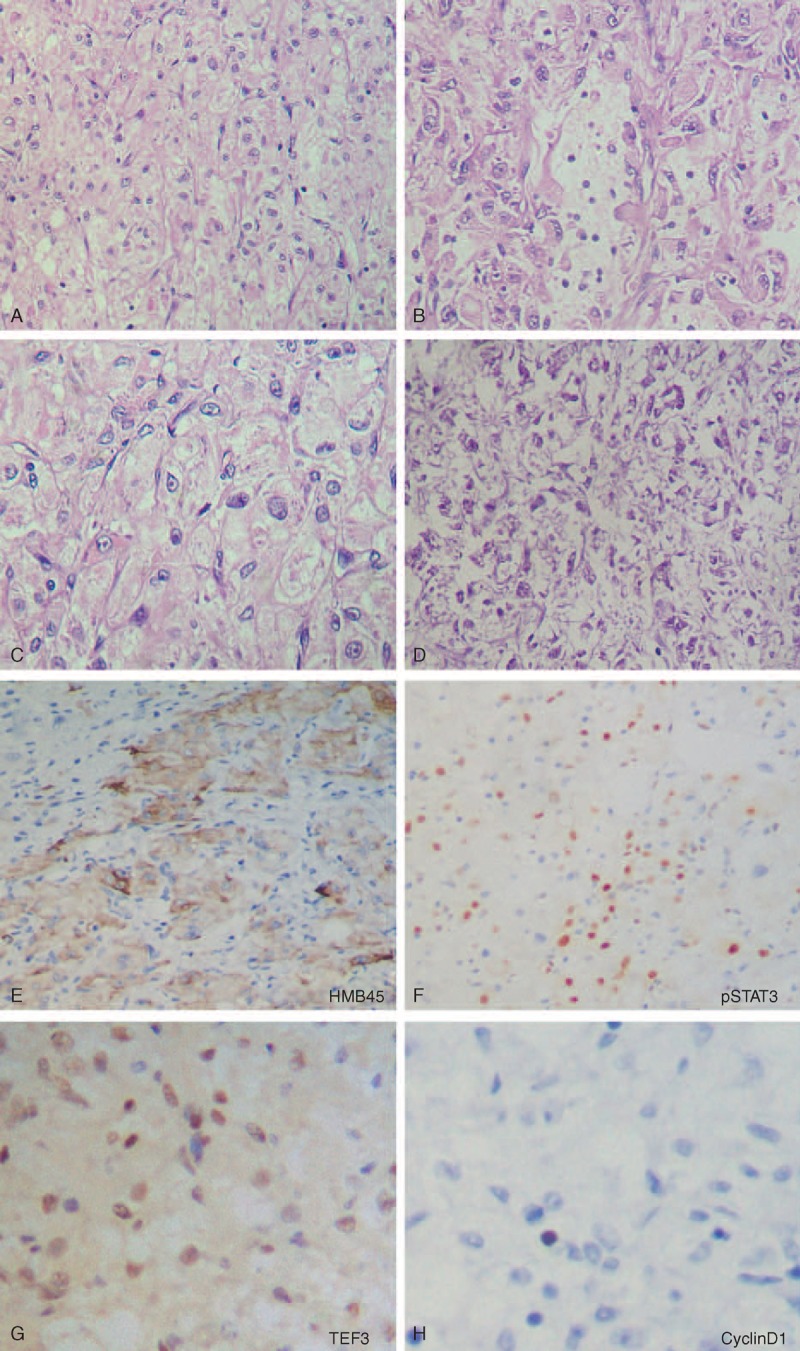
Microscopic features of the ileum tumor (case 1). The tumor consisted of an epithelioid cell proliferation with a vaguely nested pattern (A). In some areas, the tumor displayed a pseudoglandular histological appearance (B). The perivascular epithelioid cells had clear to eosinophilic granular cytoplasm with some slightly irregular nucleus nuclei (C). Foci of coagulation necrosis were also found in the tumor (D). The tumor cells were positive for HMB45 (E), pSTAT3 (F), and TFE3 (G), and negative for Cyclin D1 (H).

On gross examination, an ill-defined tumor measuring 13.5 × 7.5 × 7.0 cm was found at the terminal ileum about 12 cm from ileocecal valve (Figure 3). The cut surface showed a pinkish-grey solid parenchyma, with several scattered and irregular cystic spaces containing clear colorless serous fluid. The tumor mainly involved the muscularis propria, and protruded into the tunica adventitia, while the mucosa and submucosa were still intact. The right pelvic side wall and mesentery of the ileum were invaded by the tumor. Regional lymph nodes and lymphovascular invasions were void of any tumor. Microscopically, the tumor showed epithelioid cell proliferation with a vaguely nested pattern (Figure 4A). The nests were separated by thin fibrovascular septa. Some areas showed a pseudoglandular histological appearance (Figure 4B). The tumor cells had clear to eosinophilic granular cytoplasm. Some slightly irregular nuclei with scattered prominent nucleoli were focally present (Figure 4C). Foci of tissue necrosis and occasional mitoses (3∼5/50HPF) were found in the tumor (Figure 4D). Immunohistochemically, the tumor cells were positive for HMB45, TFE3, and pSTAT3 (Figure 4E-4G), whereas all other markers tested were negative (Table 1).

#### Case 2

**FIGURE 5:**
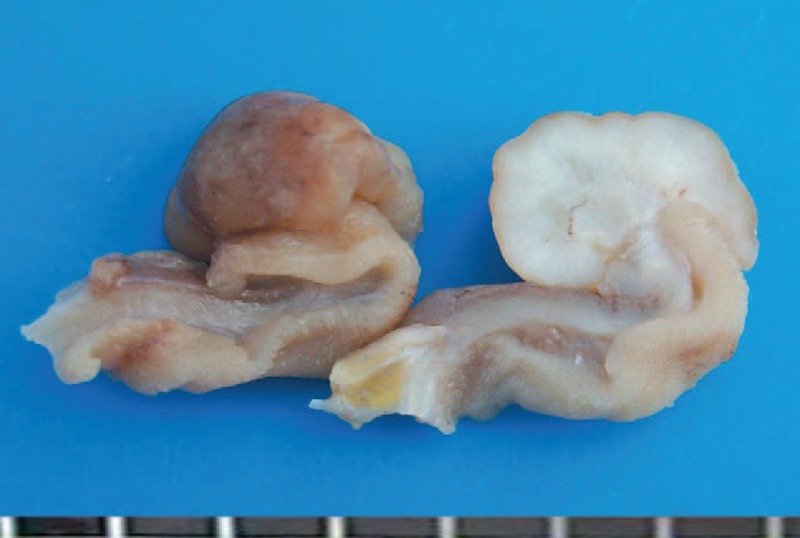
Cross-section of perivascular epithelioid cell tumor arising in the terminal ileum (case 2).

**FIGURE 6:**
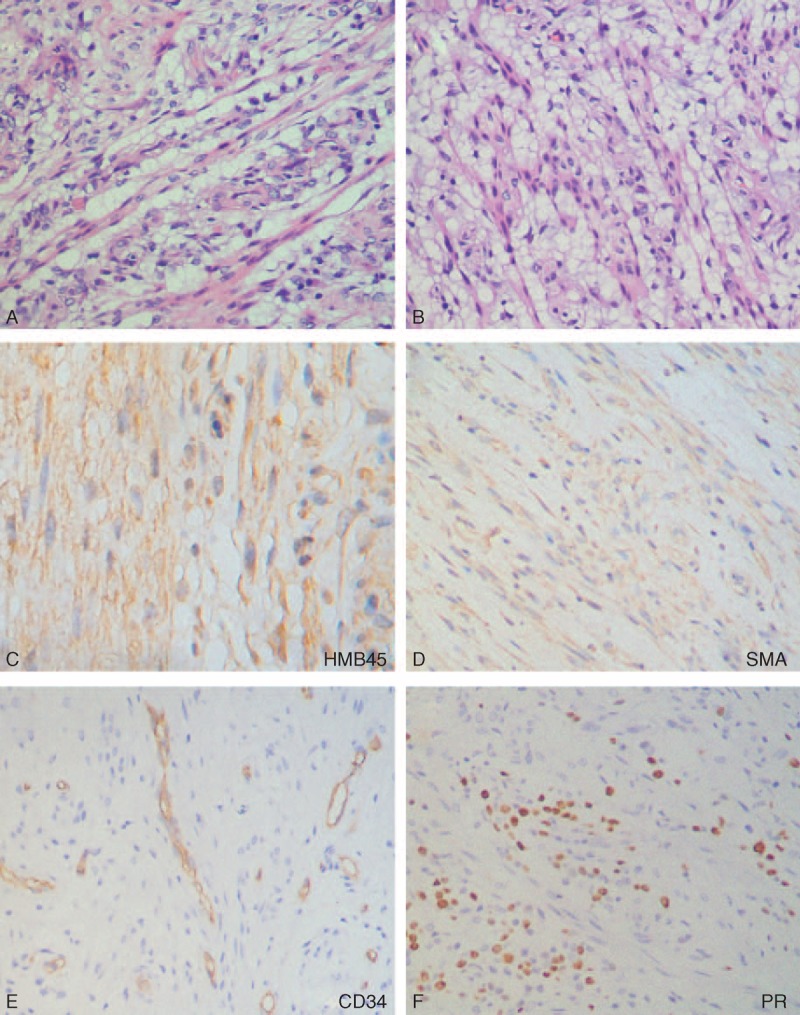
Microscopic features of the ileum tumor (case 2). The tumor showed clear spindle-shaped cells arranged in fascicular and nesting patterns (A and B). The tumor cells expressed HMB45 (C), SMA (D), and PR (F). SMA = smooth muscle actin, PR = pathogenesis-related protein

Grossly, a 2.2-cm diameter, polypoid, submucosal tumor was located in the terminal ileum protruding into the lumen. On cut section, the tumor was well-circumscribed but not encapsulated (Figure 5). Histologic examination revealed clear spindle-shaped cells arranged in fascicular and nesting patterns, separated by thin fibrovascular septa (Figure 6A-B). The nuclei were mainly round and vesicular. Neither mitosis nor necrosis was identified in the tumor. Immunohistochemical analysis revealed that the tumor cells expressed HMB45, Melan-A, SMA, HHF35, Desmin, and pathogenesis-related protein (Figure 6C–F), and were negative for all other markers tested (Table 1).

The above pathologic findings in our cases confirmed a final diagnosis of GI PEComas-NOS.

### Electron Microscopy

**FIGURE 7:**
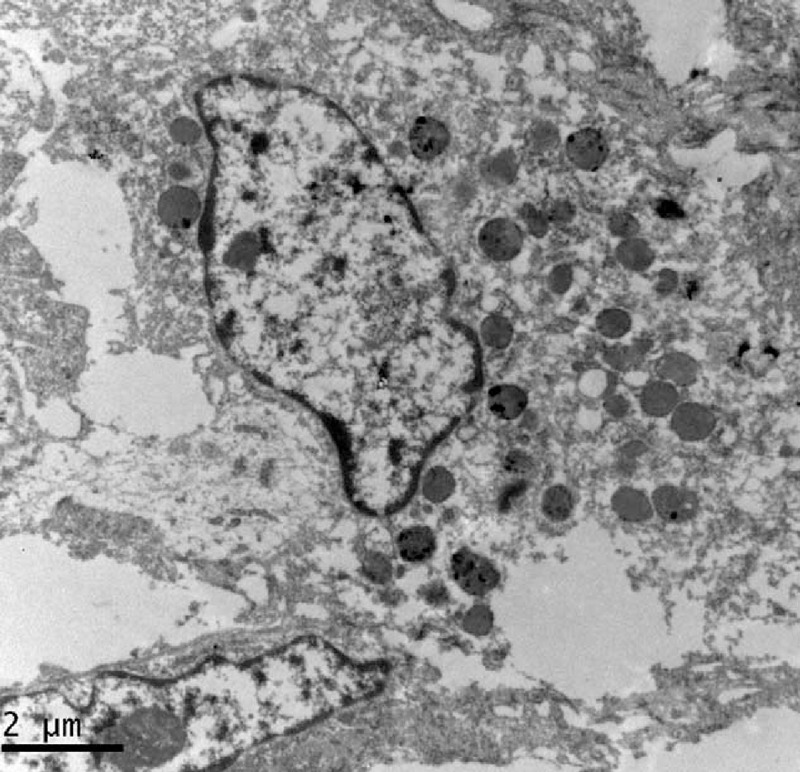
Transmission electron microscopy showing melanosomes and premelanosomes (case 1).

Melanosomes and premelanosomes were identified in the cytoplasm of both cases, using electron microscopy (Figure 7).

### Molecular Pathological Studies

**FIGURE 8:**
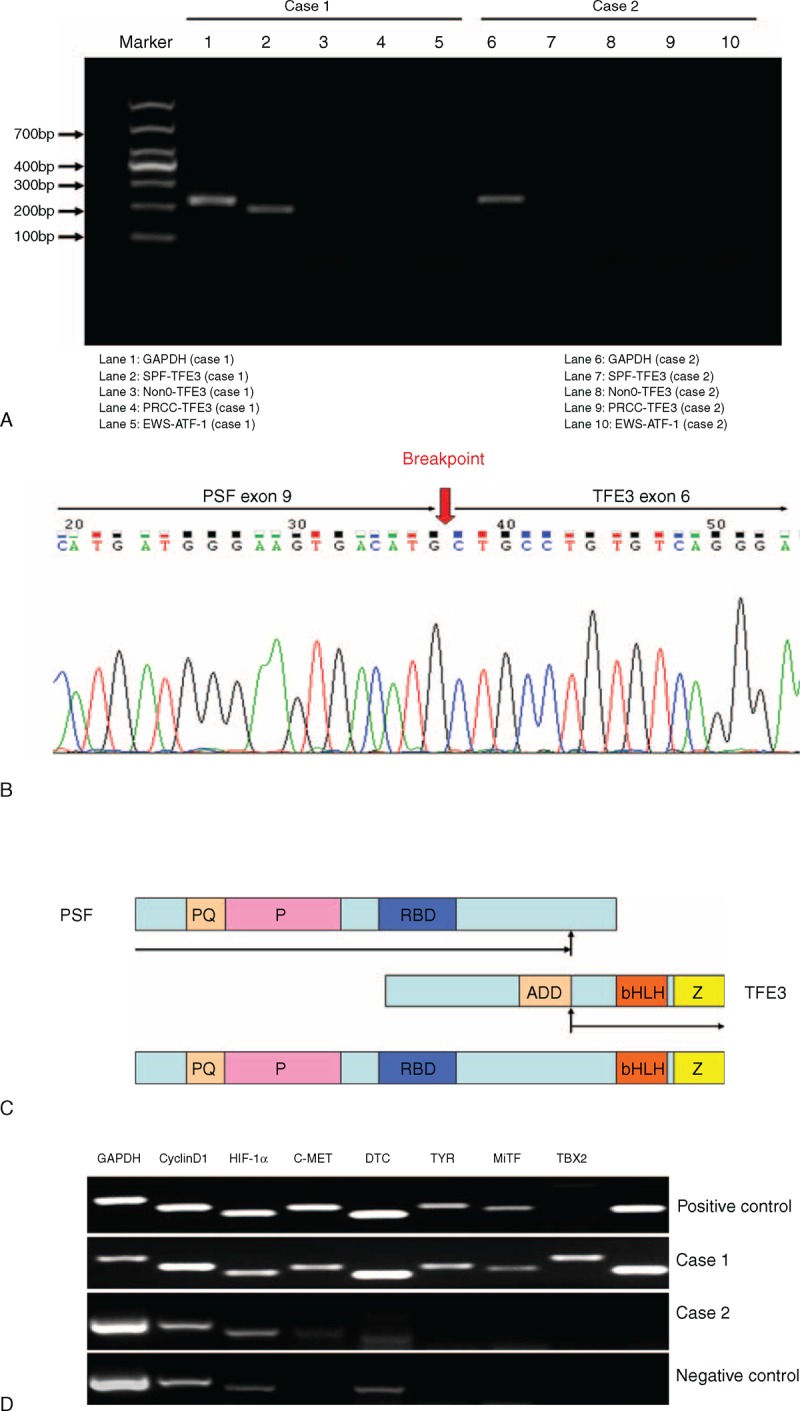
Molecular pathological analyses for PEComas. Detection of the related gene fusion fragments was performed by RT-PCR. A distinct band of 186 bp in length (lane 2) was amplified in case 1 (A). DNA sequencing demonstrated that the transcript was composed of fusions of exon 9 of the *PSF* gene to exon 6 of the TFE3 gene (B and C). Semiquantitative RT-PCR was performed to analyze the expression levels of MiTF, TYR, C-MET, DTC, TBX2, CDK2, and Cyclin D1. MiTF, TYR, CDK2, TBX2, and C-MET were up-regulated in the tumor sample of case 1 (D). PEComa = perivascular epithelioid cell tumors; RT-PCR = reverse transcription-polymerase chain reaction.

An RT-PCR assay was performed to detect related gene fusions, including *PSF-TFE3*, *NonO-TFE3*, *PRCC-TFE3*, and *EWS-ATF-1*.^4^ A distinct band of 186 bp in length (lane 2) was amplified in case 1, representing the *PSF-TFE3* fusion fragment (Figure 8A). Sequencing of the amplified DNA revealed that the *PSF-TFE3* fusion transcript contained an in-frame junction of exon 9 of the *PSF* gene to exon 6 of the TFE3 gene (Figure 8B and C). Analyses regarding the expression levels of MiTF and its downstream genes showed that MiTF, TYR, CDK2, TBX2, and C-MET were upregulated in the tumor sample of case 1 (Figure 8D). Case 2 was also tested here,, but no fusion genes were found.

### Literature Review

English-language medical reports on GI PEComas-NOS were searched for on PubMed and Embase using the search terms PEComa and gastrointestinal tract or bowel. Their clinicopathological features were reviewed and tabulated in Table 3.^1,5–30^

**TABLE 3:**
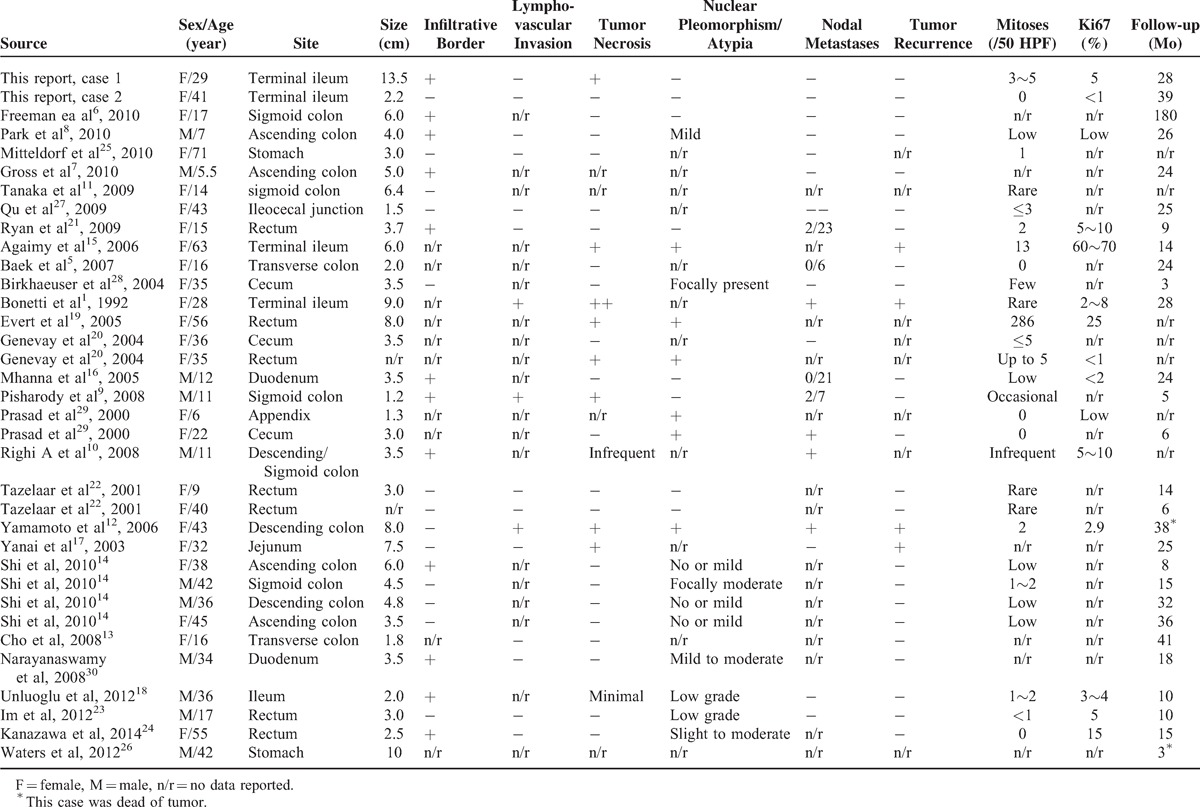
Overview of the Published Cases of GI PEComas

## DISCUSSION

According to the World Health Organization Classification of tumors, PEComas are defined as a family of rare mesenchymal neoplasms histologically and immunohistochemically characterized by PEC differentiation.^31^ The PEComa family includes AML, CCST, LAM, CCMMT, and unusual clear cell tumors in other locations.^32–35^ The latter subgroup, which has been collectively classified as PEComas-NOS, represents a collection of unusual, histologically and immunohistochemically distinctive tumors arising at various anatomic sites such as the uterus, gastrointestinal tract, and soft tissue.^31,35^ The GI tract is the second most common site of PEComas-NOS, accounting for 20% to 25% of all PEComas-NOS cases. To the best of our knowledge, only 70 cases (including the current reports) of GI PEComas-NOS have currently been reported in the English language medical literature (Table 3). The most common location of GI PEComas-NOS was the colon (n = 32),^5–14,36^ followed by the small intestine (n = 20),^1,15–18,36,37^ rectum (n = 7),^19–24^ and stomach (n = 4).^25,26,36^ Of the patients, 43 were females and 27 were males, with a ratio of 2:1. The age at diagnosis ranged from 5.5 to 71 years. Until now, all reported cases of GI PEComas-NOS were sporadic, and only 1 patient was associated with tuberous sclerosis syndrome. Given the rarity of GI PEComas-NOS and their relatively short follow-up periods, our current knowledge of their biologic behavior, natural history, criteria for malignancy, and prognostic factors is limited.

From recent clinical data, it appears that GI PEComas-NOS exhibit a spectrum of biologic behavior from benign to malignant. The majority of reported GI PEComas-NOS were considered to be benign or to have uncertain malignant potential, whereas only 22 cases (22/70) exhibited definite malignant behavior, with local recurrence in 4 cases, metastasis in 19 cases, and tumor-related death in 7 cases.^1,9,10,12,15,17,21,26,29,36^ The proposed histologic features indicative of malignancy or high risk for aggressive clinical behavior in GI PEComas-NOS by Folpe et al^38^ include infiltrative growth pattern, tumor size (>5 cm), high nuclear grade, tumor necrosis, high mitotic activity (>1/50 HPF), and lymphovascular invasion. A recent case series study of 35 GI PEComas has shown that malignant behavior was statistically significantly associated with marked nuclear atypia, diffuse pleomorphism, and mitoses ≥2/10 HPF, but not with tumor necrosis.^36^ Optimal treatment strategies for GI PEComas-NOS have not yet been well established. Currently, surgical resection with a wide margin seems to be the mainstay of treatment. The benefit of adjuvant chemotherapy,^21^ radiation, and immunotherapy^8^ has not yet been established. In case 1, the tumor showed features of malignancy in the form of large size (13.5 × 7.5 × 7.0 cm), surrounding tissue invasion, necrosis, and high mitotic activity (3∼5/50 HPF). Given the high risk of tumor aggressive behavior, the patient was treated with multiple combined chemotherapies after surgery on the basis of nonrhabdomyosarcoma soft tissue sarcoma protocol (COG-ARST0332). She was alive and well with no signs of recurrence or metastasis for 28 months of follow-up. However, the benefit of adjuvant chemotherapy is still an area of controversy that requires more evidence-based studies.

Knowledge about the molecular genetic alterations in PEComas is still limited. Some cases of AML, CCST, LAM, and CCMMT have been reported to be associated with tuberous sclerosis complex (TSC), a genetic disease caused by heterozygous mutations in the *TSC1* (9q34) or *TSC2* (16p13.3) genes, whereas only one of the reported cases of GI PEComas-NOS showed an association with TSC until now. However, Cyclin D1 overexpression has been detected by immunohistochemistry in 5 cases of PEComas-NOS that were either malignant or had an uncertain malignant potential.^12,21,39^ The role of Cyclin D1 in the pathogenesis and progression of PEComas is becoming an area of interest. However, no Cyclin D1 immunoreactivity was evident in the 2 current cases. In recent years, it has been reported that a distinctive subset of PEComas harbors TFE3 gene fusion.^40^ Tanaka et al^11^ reported the first case of GI PEComa-NOS with a *PSF-TFE3* gene fusion. In this study, we have confirmed the *PSF-TFE3* gene fusion in another GI PEComa-NOS (case 1). In addition, MiTF and its downstream genes including TYR, CDK2, TBX2, and C-MET were detected in elevated transcript levels. TFE3 and MiTF belong to the MiTF/TFE transcription factor family, which is believed to be involved in pivotal developmental and cellular processes in various cell types. In different human tissues, the ratio of expression of the MiTF/TFE family members is found to be unique.^41^ Translocations of these genes are implicated in the MiT translocation subgroup of renal cell carcinomas.^42,43^ Recently, TFE3 has also been found to be an efficient regulator in melanocyte differentiation and pigment production under specific pathological conditions in vitro.^44^ Also, results from several recent clinical studies support the hypothesis that TFE3 can substitute for MiTF in a subset of MiTF-negative PEComas.^10,13,38^ As previously proposed by others,^10,13,40,45,46^ the subset of tumors harboring *TFE3* gene fusions or exhibiting TFE3 immunoreactivity share distinctive clinicopathological features including relatively young age, nested/alveolar architecture, epithelioid cells with eosinophilic cytoplasm, and negative immunoreactivity for MiTF or muscular markers. Some studies also confirmed that overexpression of the TFE3 fusion protein is necessary for proliferation, migration, invasion potential, and long-term survival of UOK-145 cell lines.^47,48^ These findings suggest that TFE3 may play an important role in the tumorigenesis, and warrants further study.

In summary, we report 2 cases of PEComas-NOS arising in the GI tract, one of which was confirmed to harbor a *PSF-TFE3* gene fusion and to exhibit upregulation of MiTF and its downstream genes. Although the contribution of TFE3 to the pathogenesis and progression of PEComas-NOS remains poorly understood, the assessment of the *TFE3* gene status may be necessary for an accurate diagnosis and prognosis of PEComas-NOS.
